# A Computational Model of Visual Anisotropy

**DOI:** 10.1371/journal.pone.0021091

**Published:** 2011-06-30

**Authors:** Bart Ons, Leopold Verstraelen, Johan Wagemans

**Affiliations:** 1 Laboratory of Experimental Psychology, University of Leuven, Leuven, Belgium; 2 Department of Geometry, University of Leuven, Leuven, Belgium; Ecole Polytechnique Federale de Lausanne, Switzerland

## Abstract

Visual anisotropy has been demonstrated in multiple tasks where performance differs between vertical, horizontal, and oblique orientations of the stimuli. We explain some principles of visual anisotropy by anisotropic smoothing, which is based on a variation on Koenderink's approach in [1]. We tested the theory by presenting Gaussian elongated luminance profiles and measuring the perceived orientations by means of an adjustment task. Our framework is based on the smoothing of the image with elliptical Gaussian kernels and it correctly predicted an illusory orientation bias towards the vertical axis. We discuss the scope of the theory in the context of other anisotropies in perception.

## Introduction

In a variety of tasks with stimuli presented at different orientations, like line orientation discrimination, e.g. [2], line vernier acuity and contrast sensitivity, e.g. [3], [4], vertical and horizontal orientations have shown an advantage in performance compared to oblique orientations. Visual anisotropy, the unequal perception of congruent but different orientated stimuli, is a well-established finding. In the context of line orientation perception, it has been termed the “oblique effect", emphasizing the fact that the vertical and horizontal directions are discriminated better than the oblique ones [5]–[11]. However, for particular kinds of stimuli, oblique orientations are better seen than horizontal ones [12]. Although many studies have studied visual anisotropic perception and its neuronal correlates [13]–[15], it is still unclear how visual anisotropy can be conceived mathematically as a fundamental aspect of image processing. Here, we present a mathematical description of the anisotropic visual sensation through a convolution with anisotropic Gaussian kernels. We tested the theory against behavioral measurements of orientation biases using a simple stimulus that seemed to induce an illusory orientation offset. In the current report, we will first present the stimuli that seemed to induce an illusory orientation offset, then, we will demonstrate how this visual illusory perception of orientation can be related to anisotropy in resolution of the visual field. The finite resolution of the visual system has been modeled by Koenderink and van Doorn [1], [16], essentially, by smoothing the image with isotropic Gaussian kernels which have been assumed for practical ease and which have been used frequently from then on in computer vision science. Here, we will model the anisotropy in the resolution of the visual system by smoothing the initial image with elliptical Gaussian kernels instead, essentially, by smoothing more in the vertical direction than in the horizontal one. In the General Discussion section, we will elaborate on the proposed model on anisotropy and relate it to other issues in visual perception like symmetry detection, the “oblique" and the “horizontal" effect [see 12].

### The illusory orientation bias

**Figure 1:**
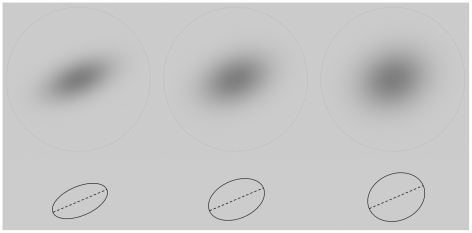
The illusory orientation bias. Three Gaussian Luminance Profiles (GLPs) are elongated in the same direction (22.5° counterclockwise from the horizontal axis). From left to right, the orientations of the luminance profiles are perceived more tilted away from horizontal as their widths increase perpendicularly to the elongated direction. Nevertheless, all stimuli have the same main orientation as demonstrated by the shape of their isoluminance contours in the lower part.

In Figure 1, three Gaussian Luminance Profiles (GLPs) are depicted. The third one seems to be tilted away from horizontal more than the first one, although the planar directions of the steepest and weakest luminance decays are identical for all three stimuli (corresponding with 22.5° counterclockwise and 112.5° from the horizontal, respectively). The only difference between the three stimuli is the steepness of the luminance decays perpendicularly to the main and most elongated directions of the Gaussian blobs. This is a non-trivial observation because when considering the luminance densities, the most natural source of information about orientation is related to the principal components of the elliptical density profile (the longest principal axes are depicted in the lower half of Figure 1). The principal axes all have identical orientations and therefore one should observe identical orientations too, but, while the magnitude of the smallest principal components should merely make the extraction of orientations more difficult, it also seemed to have biased the visual observation of orientation too. In Figure 1, there is clearly a non-trivial discrepancy between the physical descriptions of the luminance densities and the observations of it by human observers, for which we sought an account in the following section.

### Anisotropy in visual resolution

The first fundamental remark in Koenderink and van Doorn's work [1], [16] is that we see images or real scenes at a finite level of resolution. To describe mathematically the observation of the image, Koenderink and van Doorn suggested to use the luminance intensity function 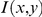 of the image, where 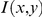 is the luminance intensity at position 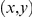 in the image plane (R^2^), smoothed with a Gaussian kernel. Numerically, the smoothed image can be obtained by the convolution 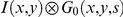, where 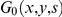 is a Gaussian with scale parameter *s*. The larger *s*, the smoother the original image will appear.

**Figure 2:**
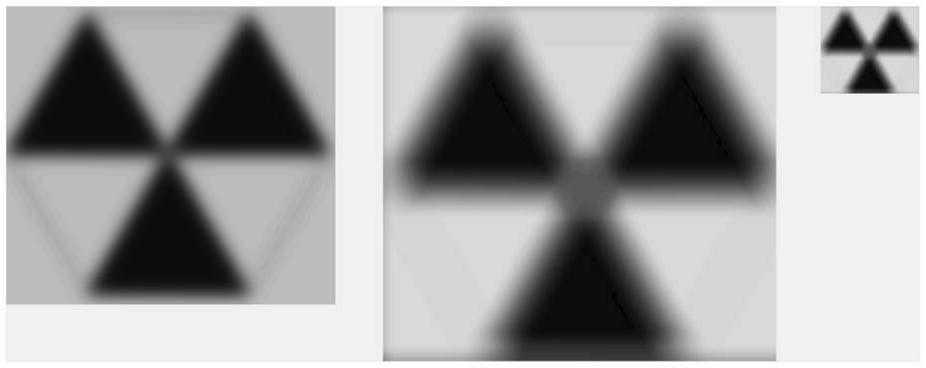
The smoothing of an image with a circular disk. (see text for an explanation).

The second fundamental remark in Koenderink and van Doorn's work is that smoothing should be done by a Gaussian kernel because it is the only smoothing procedure for which no structural artifacts are created except for the blurring. By way of example, assume that the left image in Figure 2 is smoothed by a circular disk instead of a Gaussian. The resulting image is depicted in the second image of Figure 2. By reducing the size of the second image, a smaller image appears which can be seen with more or less the same resolution as the first image (see the third image in Figure 2). One way to interpret the size-reduced image is that we are looking at the original image from a farther distance, which leads to a smaller projection on the retina. However, the second image in Figure 2 can then be interpreted as giving us a closer look on the one that is farther removed (i.e., the third image). It clearly demonstrates the new structures that appeared compared to the first one. For instance, we see a grey hexagonal center. Except for Gaussian smoothing, any other kind of smoothing will create similar spurious structures.

Koenderink and van Doorn described what they called “observation" by using convolutions to smooth images. Although the term “convolution" is widely used in computational and neurobiological models on neuronal coding, Koenderink and van Doorn seemed not to intend to theorize about neuronal coding. Similarly, in the current study, a neuronal model is not pursued. We adopted Koenderink and van Doorn's mathematical model on the finite resolution of the visual system by smoothing the image and we added a natural anisotropic retouch to their hypothesis of isotropy of the visual field. We thus hypothesized that the observed image is the result of a convolution operation with an anisotropic Gaussian kernel that is more elongated in the vertical direction compared to the horizontal direction. Our hypothesis in its most simple form involves a two-dimensional scale parameter, one in the horizontal and one in the vertical direction:
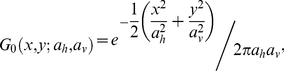
(1)

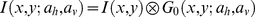
(2)where 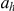 and 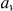 are the horizontal and vertical radii of the Gaussian aperture, respectively, and *a_h_* is smaller than *a_v_*.

The GLPs in Figure 1 are convenient to examine this hypothesis by the computational simplicity of the convolution of two Gaussians. The outcome is also a Gaussian and can be easily computed by summing the density-related covariance parameters. When the covariance matrices are denoted by 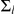
and 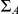 for the administered GLP and the Gaussian smoothing kernel, respectively, then the covariance matrix of the perceived Gaussian 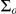 becomes:
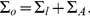
(3)


**Figure 3:**
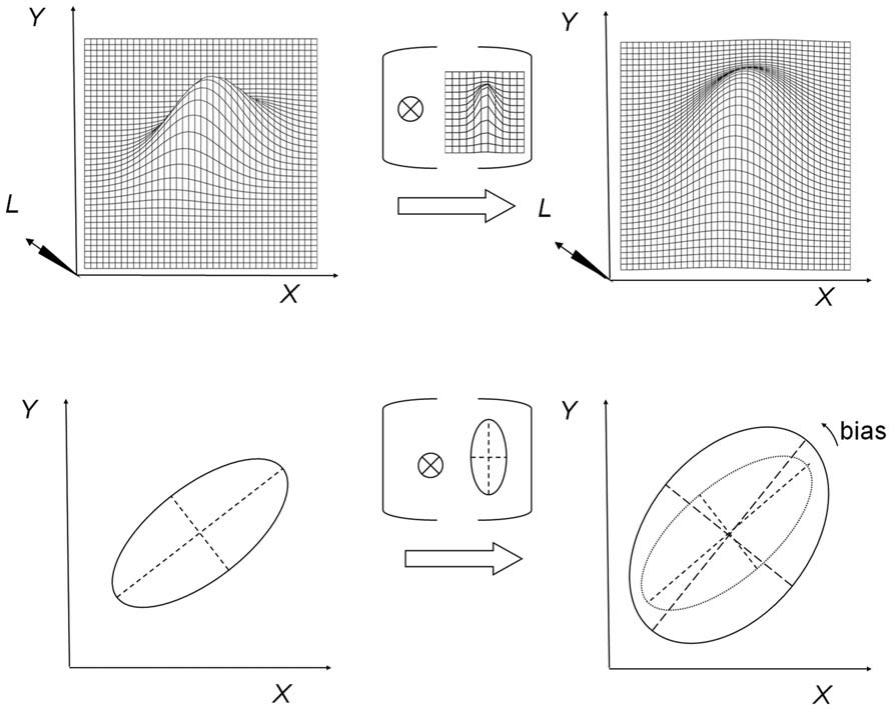
A visualization of the Gaussian convolution leading to a biased orientation perception.

A common practice in computer vision is to visualize the luminosities by a relief surface in a three-dimensional space, the image space, with two spatial dimensions *X* and *Y* forming the image plane and one physical dimension *L*, with *L* denoting luminance intensity. A relief surface can also be visualized topographically in the *XY*-pane by one or more isoluminance contours. The relief surface is depicted in the upper part of Figure 3 and the corresponding isoluminance contour is depicted in the lower part of Figure 3. The large ellipse at the left represents some administered GLP on a monitor screen. The smoothing kernel is represented here by the small relief surface between brackets (or the ellipse between brackets in the lower panel). The convolution operation will lead to the figures at the right in Figure 3. Observe in the lower panel that the major axis of the convolved relief surface (the observed GLP) is slightly rotated away from the major axis of the initial ellipse (the administered GLP). The less steep the descent of the relief surface of the administered GLP, the more the final relief surface becomes tilted away from the horizontal orientation *X*. The orientation shift will also depend on the original orientation of the GLP. A convolution with an elliptical Gaussian kernel with the horizontal radius smaller than the vertical radius can thus explain the different orientation biases in Figure 1. Note that, for instance, a simple stretch of the vertical dimension cannot explain the visual observation in Figure 1. When the *XY*-plane would be stretched in the vertical direction, then all three stimuli from Figure 1 would be subject to the same orientation shift, which is clearly not the case.

In short, our theory suggests that anisotropy is related to the limited resolution of the eye, which we hypothesize to be higher in the horizontal than in the vertical direction. Our model is based on Koenderink and van Doorn's approach of Gaussian smoothing. However, we introduced anisotropic smoothing, and, as we will further elaborate in the discussion section, this simple model does a good job in explaining and unifying many sorts of seemingly different kinds of perceptual phenomena of anisotropy.

### Aim of the experiment

The aim of the experiment is to investigate the anisotropy in the perception of GLPs. We created GLPs with different main axis orientations and different thicknesses (different lengths of the axis orthogonal to the main axis), and measured psychophysically the perceived orientation biases using an adjustment task. The experimental data was used to validate the model of anisotropic smoothing.

Note that, at this point, the elliptical Gaussian kernel in Equation (1) is still considered to be homogeneous throughout the visual field. Homogeneous refers to smoothing with the same kernel at every location in the visual field. We call this model “*Homogeneous anisotropic smoothing*". Anisotropic smoothing with kernel radii *a_h_<a_v_* leads to the expectation of an orientation bias towards the vertical direction. However, the resolution of our eye is probably different in the periphery compared to the center of the visual field. Therefore, we also considered two “*Heterogeneous anisotropic smoothing*" models. For these models we expected, for instance, to meet larger Gaussians in the periphery than in the central part of the visual field.

## Materials and Methods

### Participants

Five volunteers and the first author participated in the experiment. All participants had normal or corrected-to-normal eye sight. The authors confirm that the research has been conducted according to all ethical standards imposed by their Ethics Committee at the University of Leuven, who approved the study. Written informed consent was obtained by all participants, according to the procedures imposed and approved by the above Ethics Committee.

### Apparatus

The stimuli were presented on a 17″ Dell monitor with a resolution of 1024 by 768 pixels and an average refresh rate of 75 Hz. The PC was a Dell Optiflex Gx620, Pentium IV, 2.8 GHz. Participants' position was fixed at a distance of 40 cm from the screen by a chin rest. Participants had to indicate their responses by means of a mouse. Moving the mouse resulted in a manipulation of the orientation of a green thin line positioned on top of the GLPs. The upper left button of the mouse was used to confirm the adjustment setting and to terminate the trial.

### Stimuli

Luminance values on a monitor are discrete values and two borders between two regions of pixels with the same luminance values are clearly visible to the human eye. Therefore, we added white pixel-noise (not visible to the bear eye) to the stimuli. The luminance range of the pixel-noise was small and only consisted of 2% of the luminance range of the GLPs. The luminance of the GLPs ranged from 10% to 30% of the total luminance range in a 24 bit color depth bitmap format. Within the limited range of the GLPs, light intensities and luminance values of pixels are approximately linearly related. The luminance amplitude in the center of all GLPs was 2.70 cd/m^2^ and 1.54 cd/m^2^ in the background, measured with a Minolta Chromometer cs-100 from the eye position of the participant.

There was a dark gray circle displayed on the monitor screen that enclosed the reference stimulus (see Figure 1 for an example). The center of the circle was fixed on the center of the GLP. The radius of the circle measured 5.6° of visual angle and was large enough to enclose the GLP at a distance from the centre where no luminosities of the GLP were visible.

### The reference stimuli in the adjustment task

The reference stimuli consisted of GLPs with three parameters of physical interest: the length of the major and minor radii determining the luminance density and the orientation of the main radius (see Figure 1). While the length of the main radius was held constant at 1.9° of visual angle, the length of the minor radius was manipulated between 0.95°, 1.235° and 1.52° of visual angle (see Figure 1 for an example). The manipulation of the minor radius resulted in three aspect ratios for the GLPs: 0.5, 0.65 and 0.8. We refer to the variation in thickness by “Small GLP", “Medium GLP" and “Large GLP". The luminance profiles made a gradual transition from the center to the background.

The three thickness types of GLPs were crossed factorially with a set of reference orientations. The main orientation of the major radius was oriented counterclockwise from the horizontal axis in uniform steps of 15° along the whole periodic domain of orientation (not including the cardinal orientations) and corresponded to 7.5°, 22.5°, 37.5°, 52.5°, 67.5°, 82.5°, 97.5°, 112.5°, 127.5°, 142.5°, 157.5° and 172.5°. Note that the perceived dimension of orientation has a period cycle of 180° instead of 360° because the reference stimuli have two axes of mirror symmetry. Rotating a GLP over an angle of 180° will lead to an identical GLP.

### The probe stimulus in the adjustment task

In order to merge the probe and the reference stimulus in the same observation area, the probe line was laid on top of the reference stimulus. While all reference stimuli were gray, the color of the probe stimulus was green and consisted of a thin anti-aliased line with the same length as the diameter of the circle enclosing the reference stimulus and with its center of rotation fixed in the center of the circle and the GLP. The probe stimulus could be adjusted by the mouse in small discrete steps over the complete range of orientations. The step size between two consecutive probe orientations was 1°. The probe line followed the angular path of the mouse and gave the impression of a smooth continuous rotation around the center. Because of the movement and color of the probe stimulus, it appeared naturally as a different entity from the reference stimulus and adjustments could be carried out in a straightforward fashion, taking only a few seconds for each trial.

### Controlling for frame display

To control for the radiation emerging from the display and for the shape of the reference frame emerging from it, we used two different viewing apertures, consisting of an opening in a cardboard covering the monitor, one having a circular shape with a diameter of 8 cm just enclosing the displayed gray circle, and the other having a larger square shape with side lengths of 15 cm. The luminance measured from the non-transparent sheet enclosing the observation area was 0.07 cd/m^2^. The purpose of this control variable was to verify whether any bias effect could be modulated by the frame, i.e., amount of light radiating from the monitor (larger with the square aperture), the reflection of light in the room (larger with the square aperture) and the orientation of borders by the frame (horizontal and vertical for the square aperture). If the orientation bias would be the result of an artifact like the frame enclosing the observation area, we would expect to see a stronger (or at least a different kind) of bias for the square aperture. Irrespective of the frame aperture, there was a light gray circle displayed on the monitor screen that enclosed the reference stimulus in both conditions.

### Procedure

In the adjustment task, participants were instructed to adjust the probe stimulus to the same orientation as the orientation of the most elongated direction of the reference stimulus. There was one session for each condition of the frame display consisting of three blocks of 180 trials. In each block, each reference orientation was probed five times for each thickness, resulting in 15 trials per reference orientation and per thickness in each session. While half of the participants started with the session with the circular cardboard aperture, the other half started with the session with the squared cardboard aperture. In total, there were 1080 observations per participant.

The dependent variable was the illusory orientation bias derived from the difference between the adjusted orientation of the probe line and the physical main orientation of the reference stimulus (see Figure 3). The sign of a counterclockwise bias was chosen to be positive.

### Modeling

There were three models of interest and one reference model. The reference model called, “*isotropic smoothing*" is the most simple model assuming equal vertical and horizontal radii of the Gaussian kernel. Irrespective of the scale, no orientation bias can be expected. A larger scale will lead to more blurring but GLP's main axis orientations cannot change. The isotropic Gaussian kernel model is nested in the first model of interest, the “*Homogeneous anisotropic smoothing*" model, in which we allowed the horizontal and vertical radii to be different from each other. This model is nested in the second model of interest, the “*Radial anisotropic heterogeneous smoothing*" model. Heterogeneous anisotropic smoothing allows for the variation of the horizontal and vertical Gaussian kernel radii across the visual field. On average, this will lead to a different smoothing for each GLP. Heterogeneous anisotropic smoothing with different kernel parameters in each possible location of the visual field leads to a very complex modeling procedure. Therefore, we evaluated heterogeneity in a different manner. We divided the GLPs in subsets and estimated the best kernel parameters for each subset assuming homogeneous smoothing within each subset. If homogeneous anisotropic smoothing is the “true model", then all estimated parameters for each GLP subset should be the same. However, when larger kernels are to be expected in the periphery of the visual field, we can expect differences in estimated parameters for the subsets comparing the larger GLPs with the smaller GLPs. In the first heterogeneous model, we estimated *a_v_* and *a_h_* separately for each subset of equal GLP thickness. A significant improvement for the latter model compared to the anisotropic homogeneous model leads to the conclusion that smoothing should be considered heterogeneous. Similarly, we can divide the GLPs in subsets of equal orientations and verify the angular component of heterogeneity. When kernels are heterogeneous with respect to the angular component of the visual field, we can expect a significant improvement for estimating *a_v_* and *a_h_* separately for each subset of GLP orientation. In the “*Angular heterogeneous anisotropic smoothing*" model, we followed this procedure. Additionally, we also incorporated a third parameter *r* related to the covariance of the kernels. For the anisotropic homogeneous and the radial heterogeneous anisotropic model, *r* was assumed to be zero and kernels were therefore horizontal-vertical oriented. By taking into account the covariance of the kernel's density matrix, the kernels are allowed to have a proper orientation. A significant improvement of the latter model, compared to the homogeneous anisotropic smoothing model leads to the conclusion of heterogeneity in the angular direction of the visual field. The major advantage of this procedure is that the anisotropic homogeneous model is still nested in the two anisotropic heterogeneous models.

When one model (the more restricted one) is nested within a second model (the less restricted one), we can firmly compare the two models by an *F* test: 
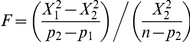
 where 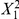 is the weighted residual sum of squares of the more restricted model, 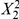 is the weighted residual sum of squares of the less restricted model, 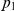 and 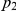 are the number of estimated parameters for the more and less restricted models respectively and *n* is the number of data points. Under the null hypothesis that the less restricted model does not provide a significantly better fit than the more restricted model, *F* will have an *F* distribution, with 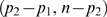 degrees of freedom. For each participant, the homogeneous anisotropic model has 2 free parameters (vertical and horizontal kernel radii), the radial heterogeneous anisotropic model has 6 free parameters (vertical and horizontal kernel radii for each thicknesses) and the angular heterogeneous anisotropic model has 36 free parameters (vertical, horizontal radius and orientation of the kernel for each GLP orientation).

## Results

The frame display variable was introduced as a control variable and we did not find an effect of frame display. The data was therefore pooled over both conditions of the frame display, resulting in 30 repeated measurements per reference orientation, per thickness and per participant. In the analysis, we will focus on the relation between the thickness of the reference stimuli, the reference orientations and the perceived orientation biases.

From a visual inspection to the data, we found that the spread of responses was rather normally distributed but the magnitude of the variances differed for the different thicknesses of the GLPs. Therefore, in all models, we fitted *a_h_*, and *a_v_* by the method of weighted least squares with the inverse of the variances constituting the weight matrix.

**Figure 4:**
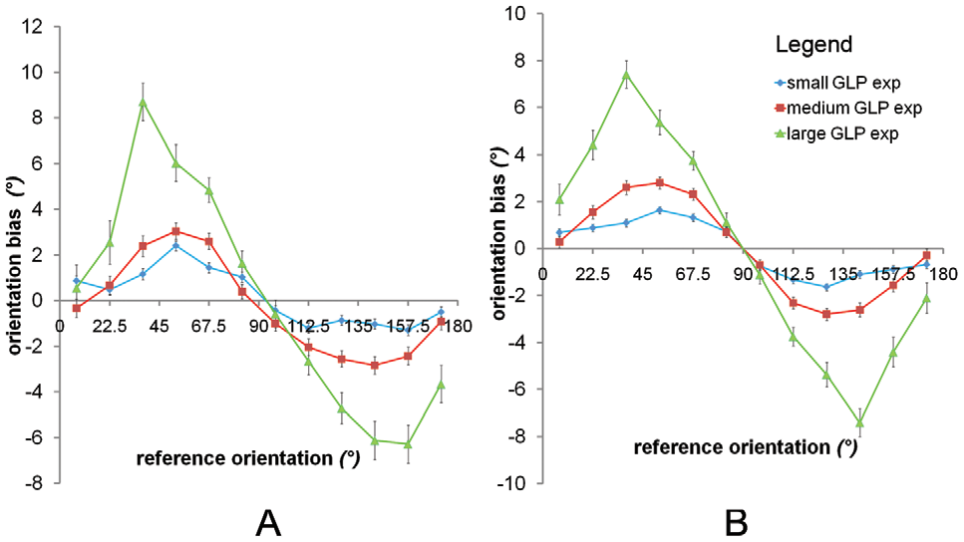
The results from the psychophysical experiment. A) showing average orientation biases (the vertical axis) for all twelve reference orientations of the GLPs (horizontal axis) and each thickness of the GLPs (colors, see legend). The bias is denoted as the difference between the perceived orientation and the real orientation of the administered GLP. B) A regularized version of the data less vulnerable to overfitting is shown. The bias magnitudes are averaged over pairs of orientations under the assumption that the bias magnitudes should be equal for the mirrored GLPs over the vertical axis. Error bars denote SEMs.

In Figure 4A, the average biases for all participants are plotted for each condition. The figure shows a positive (counterclockwise) bias for GLP orientations proceeding counterclockwise from the horizontal to the vertical direction (from 7.5° to 82.5°) and a negative bias (clockwise) proceeding counterclockwise for the GLPs from the vertical to the horizontal direction (from 97.5° to 172.5°). Overall, the biases always tended towards the vertical orientation.

In Figure 4A, biases showed many fluctuations and it is difficult to determine whether these fluctuations represent random noise or a causal signal. On the one hand, neglecting these assumed random fluctuations might lead to a simple model missing some essential aspects of the mechanisms underlying the data. On the other hand, by taking data fluctuations falsely for a real signal, models might overfit the data and predict the random variations by spurious parameters that just happen to help the model's predictions to jump up and down along these fluctuations. In view of the current data, we believed that risk of overfitting was more severe, and therefore, we regularized the data. We accepted the hypotheses that the bias magnitudes should be the same for the GLPs that have the same orientation when they are reflected across the vertical axis of the monitor screen (i.e., 7.5° and 172°, 22.5° and 157.5°, 45° and 135° and for 67.5° and 112,5°). By assuming this symmetry between the respective orientations, the experimental data can be aggregated for orientation pairs, and the data obtained from this procedure is plotted in Figure 4B. The experimental data show much less fluctuations and the chance of overfitting the data becomes smaller. The reported models in the current study are tuned to the aggregated data presented in Figure 4B. By aggregating the data over pairs of GLP orientations, only 6 orientations (instead of 12) are remaining, and the number of free parameters of the angular heterogeneous anisotropic model reduced from 36 to 18.

### Homogeneous anisotropic smoothing

**Figure 5:**
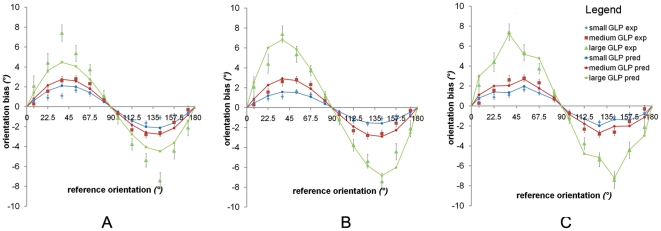
Model predictions. The three trend lines (see legend) are the average predictions per GLP thickness for the six participants for A) homogeneous anisotropic smoothing, B) radial heterogeneous anisotropic smoothing (*a_v_*, *a_h_* depending on GLP thickness) and C) angular heterogeneous anisotropic smoothing (*a_v_*, *a_h_*, *r* depending on GLP orientation). The markers are the average experimental results adopted from Figure 4B.

Here, we compare anisotropic against isotropic smoothing. The estimated Gaussian kernel radii with *a_h_*<*a_v_* led to a significant improvement in the prediction of the biases for each participant (for all participants, *F*_(2,1078)_>18, p<0.0001). The predictions are depicted in Figure 5A. The estimated kernel radii were similar between participants, *a_h_* = 0.84 (sd = 0.50) and *a_v_* = 2.08 (sd = 0.21). The explained variance seemed to be rather low (13.4%, 18.9%, 11.2%, 3.3%, 21.2%, 3.5% for each participant, respectively). There was a lot of response variability due to the nature of the stimulus. It is not easy to extract a dimension like orientation from a Gaussian luminance profile. Additionally, the explained variance is considerable when we compare the explained variance against the systematic explained variances, which are maximal obtainable variances given the noise in the data (eliminating the variance within each condition); they are 19.8%, 23.3%, 16.1%, 11.2%, 27.2% and 5.8% for each participant, respectively. Clearly, there seemed to be a lot of noise in the data confirming our presumption that the fluctuations in Figure 4A were probably due to random noise. A second reason explaining the low explained variance is the restriction to predict orientation biases only through a homogeneous Gaussian kernel, i.e., one kernel with constant aperture radii for the whole visual field. We will investigate whether the less restricted heterogeneous anisotropic smoothing models provide better estimations to the data.

### Radial heterogeneous anisotropic smoothing

This model only serves to answer the question whether smoothing should be considered homogeneous or not. As explained in the Methods section, a significant improvement for estimating different kernels for different subsets of GLPs gives the answer to this question. However, the estimation of the kernel parameters should not be interpreted as a quantitatively veridical analysis of kernel parameters at different positions in the visual field.

Statistically, for five out of six participants, different smoothing radii for each subset of GLP thickness led to significant improvements compared to homogeneous anisotropic smoothing (all five participants had *F*_(4,1074)_>3.39, p<0.01). For the participants where we found the strongest improvements (Participant 1, 2, 3 and 5), the length of the horizontal radii, and in accordance the anisotropy, seemed to increase slightly for thicker GLPs. The explained variance improved slightly (16.8%, 20.4%, 14.6%, 4.5%, 23%, 3.6%, respectively). The predicted values are depicted in Figure 5B.

### Angular heterogeneous anisotropic smoothing

While heterogeneous anisotropic kernels involved different radii for each thickness, but still the same radii for each orientation, we will now implement a model by estimating different radii for GLP subsets with the same orientation, but different thicknesses. Additionally, for each orientation, we will also include a parameter to estimate the orientation of the main axis of the smoothing kernels. We compared angular heterogeneous anisotropic smoothing with the nested and more restricted model of homogeneous anisotropic smoothing. However, to have a fair comparison between both models, we also allowed the same orientation-dependent parameter in the model of homogeneous anisotropic smoothing leading to three free parameters for the restricted model. Similarly to the former radial heterogeneous model, we found a significant improvement for the same five participants (*F_(15,1062)_>2.29, p<0.005*). The explained variances were in the same range as in the former heterogeneous model. The predicted values are depicted in Figure 5C. As expected, the estimated orientation of the kernel in the homogeneous anisotropic smoothing model was vertical. However, the orientation of the estimated kernels main axes in the angular heterogeneous anisotropic smoothing model was more or less perpendicular, slightly more vertical, to the orientations of the GLP subsets. The anisotropy and the size of the kernels increased for the oblique oriented GLP subsets.

### Modeling the dispersion of orientation responses

**Figure 6:**
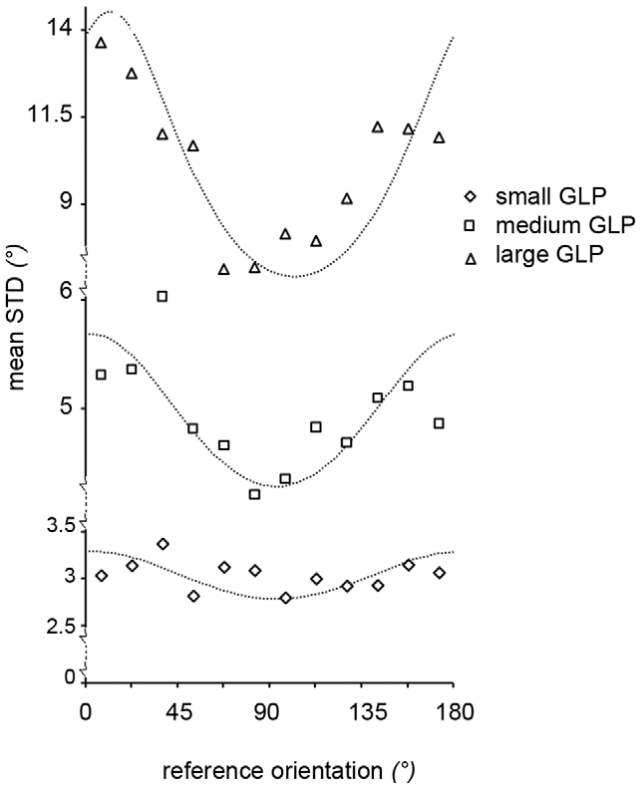
Mean standard deviations. Average standard deviations of the orientation adjustments are plotted for each orientation and thickness. The orientation adjustments were more concentrated for vertically oriented GLPs (90°) and more diffuse for horizontally oriented GLPs (0° or 180°).

There was a lot of noise in the data. However, the response variances showed a clear pattern that can be expected from the current model. The thicker a GLP, that is, the more roundish a 2D Gaussian, the more difficult it becomes to adjust the orientation of a line to the orientation of the GLP, and the more variance that can thus be expected. For the smoothing with a vertically oriented kernel, the horizontally oriented GLPs become more roundish than the vertically oriented GLPs, leading to more diffuse orientation responses for the horizontally oriented GLPs. In Figure 6, we plotted the standard deviations of the orientation responses averaged over participants for each thickness and for each orientation separately. Clearly, the pattern of response variability in Figure 6 is showing more dispersion for the more horizontal orientated GLPs than for the vertical oriented GLPs. It can be considered as a further verification of the models on anisotropy, essentially, anisotropy by smoothing the image more vertically than horizontally. In the light of the “oblique effect", the pattern of response variability in Figure 6 is counterintuitive. According to the “oblique effect" hypothesis, larger variances for obliquely orientated GLPs can be expected but not for horizontally orientated GLPs.

## Discussion

In the present study, we introduced a new, rather subtle horizontal-vertical illusion. The main orientation of an elongated luminance profile with smooth boundaries is perceived to be more vertical tilted than what can be presumed from the physical angular coordinates. A subtle difference in thickness of the luminance profile seemed to induce a strong effect on the magnitude of the perceived orientation bias. These findings were consistent with the predictions from the proposed models on visual anisotropy, i.e., to consider the finite resolution of the eye by smoothing the original image with Gaussian elliptical kernels for which the major axis was rather oriented vertically. Moreover, based on the modeling results, there is a strong indication that the Gaussian aperture is also heterogeneous at different positions in the visual field. The kind of stimuli and the method of adjustment seemed to have provided a useful tool to measure particular aspects of resolution across the visual field. Anisotropic smoothing can shed some light on different perceptual phenomena like, for instance, symmetry detection and the oblique and horizontal effect. We will discuss its implementation for these domains separately.

The theory of visual anisotropy can play some role of importance for symmetry detection [17]–[20]. It has been found that mirror symmetry is easier to detect for shapes or dot patterns when the symmetry axes are oriented vertically or horizontally instead of obliquely. In all proposed models, the shape of a symmetric figure becomes affected by smoothing. The smoothed image will not preserve symmetry when the axis of orientation is obliquely oriented. Only for a horizontal or vertical axis of symmetry, the smoothed figure will preserve symmetry. When the axis of symmetry of the figure is oriented vertically or horizontally, both symmetric parts of the figure are smoothed or blurred in the same way, namely corresponding luminosities at both sides are multiplied with the same Gaussian kernels. Inversely, when the orientation of the symmetry axis in the image is obliquely projected on the retina, then corresponding points at the boundaries at both sides of the symmetry axis will be multiplied with different Gaussian kernels, and therefore, a subtle difference in the shape of the smoothed luminance intensity function will be induced at corresponding locations at both sides, essentially, leading to a degradation of symmetry.

The “oblique effect" is a well-established finding and most reported results confirmed the superiority of the vertical and horizontal directions, being perceived more precisely than the oblique ones [5]–[11]. The current models can shed some light on this phenomena. Based on the parameter estimations of the heterogeneous models, kernels are estimated to be smaller in size for vertically and the horizontally oriented stimuli leading to better discrimination for horizontal and vertical lines than for oblique ones. Consequently, there is a higher resolution along the cardinal axes of the visual field.

**Figure 7:**
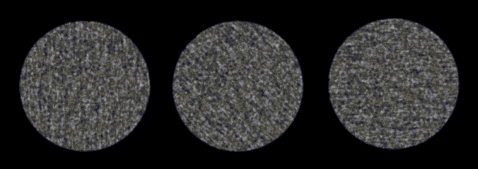
Anisotropic stimuli with a particular oriented texture. For most observers and also depending on the distance from the page, the oriented tracks are best perceived in the middle (oblique) and the left (vertical) stimulus, then in the right stimulus (horizontal). Actually, all stimuli are identical, except for a rotation.

The oblique effect (which is related to the discrimination of orientations) is also strongly depending on the kind of stimulus as well. For instance, contrary to a sharp line the orientation of smooth stimuli like the current GLPs (see Figure 6) seem to be discriminated more consistently for oblique and vertical orientations than for horizontal ones. Similar to our smooth GLPs, the superiority of oblique and vertical orientations (termed the “horizontal effect") has been reported in Essock, Deford, Hansen, and Sinai [12]. In their study, isotropic stimuli were created, and subsequently, manipulated in the Fourier domain to obtain a stimulus similar to the ones presented in Figure 7. The initial isotropic stimuli were actually smoothed more in one particular directions to create experimental stimuli with an oriented structure in the same direction as the smoothed one. By smoothing in one direction, the nods and the dots become more connected leading to the impression of a particular track orientation. Participants were better able to detect the obliquely and vertically oriented structures (middle and left globe in Figure 7) than the horizontal one (right globe). Actually, all globes are identical except for a rotation. Turning the page 45° counterclockwise will affect the ranking with the worst visible track orientation in the middle globe, then having a horizontal orientation. From the current model, the “horizontal effect" can be explained straightforwardly. A particular track direction is created beforehand by smoothing some isotropic image (like randomly located circular blobs) more in one particular direction. When the orientation of the globe tracks are created by smoothing more in the horizontal direction, and secondly, when the resolution of the eye is rather characterized by smoothing the image in the vertical direction, the final result (horizontal smoothing+vertical smoothing) will lead to the perception of a more blurred, but also more isotropic image. The orientation of the tracks become relatively more difficult to see. In contrast to the horizontally smoothed experimental images, the vertically and obliquely smoothed experimental stimuli with vertically and obliquely oriented tracks, respectively, are smoothed further in the same directions due to the characteristic resolution of the eye. The tracks in the perceived images become even more smoothed anisotropically (vertical smoothing+vertical smoothing) and the direction of the tracks becomes even more visible.

Clearly, anisotropic smoothing defined by convolving the image with anisotropic Gaussian kernels, can explain some psychological phenomena of anisotropic visual perception. However, our model does not include a technical implementation of the smoothing procedure in the brain. Technically, there are different possibilities to implement this kind of smoothing, for instance, by populating more vertically oriented elliptically shaped Gaussian components in the elementary Gabor filter models of V1, by reducing the frequency amplitudes in the Fourier domain of the orientation sensitive neurons for one particular direction, or by taking the optics of the eye (refraction of light, the shape of the retina, etc) into account. A neuroscientist would probably prefer to implement this kind of smoothing in the Fourier domain, for instance, by an overall reduction of sinusoidal frequency amplitudes of the elementary Gabors in the vertical direction. Actually, whether smoothing luminance values of the luminance intensity function 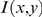 or reducing frequency amplitudes in a particular orientation of the same function transformed to the Fourier domain, both kinds of technical implementation are equivalent to each other and should be considered as two possible treatments of the same subject matter. We did not intend to theorize about the technical implementation of our model, but, the current mathematical model shows that all technical implementations leading to a lower resolution in the vertical direction of the visual field would probably do a good job in explaining many experimental data of multiple anisotropic visual phenomena [21].

In the introduced model of visual anisotropic resolution, the physical image is convolved with elliptical Gaussian kernels leading to a smooth luminance profile at the cost of a more blurry image. Naturally, the conscious experience of human observations is not that of a blurry external world but rather one of colored surfaces with high contrasting boundaries. Physical images, kernels and luminance profiles do not exist in the “real" world, but only in theoretic thoughts of the mind by which we try to describe, understand and unify some phenomena. Theoretically, we do not see a blurry image because visual processing does not stop with the smoothing of light entering the eye. The current model describes only one step in visual processing, the one that makes luminance reliefs smooth and differentiable. The smoothing should rather be considered to form one of the first steps in visual processing. From there on, different measures on the smoothed image surface can lead to a percept corresponding more to the experience of colored surfaces with high contrasting boundaries. Although the luminance profile is smooth in the image space, some operations related to the curvature of the luminance surface and the corresponding extrema can, for instance, extract sharp boundaries [22]–[25]. The proposed theory in combination with the simple stimuli is a fruitful approach to describe mathematically one of the first steps in visual perception: the anisotropic sensation of luminance.
